# Strain-Rate Effects on the Mechanical Behavior of Basalt-Fiber-Reinforced Polymer Composites: Experimental Investigation and Numerical Validation

**DOI:** 10.3390/ma18153637

**Published:** 2025-08-01

**Authors:** Yuezhao Pang, Chuanlong Wang, Yue Zhao, Houqi Yao, Xianzheng Wang

**Affiliations:** 1 Marine Design and Research Institute of China, Shanghai 201203, China; 2 College of Aerospace and Civil Engineering, Harbin Engineering University, Harbin 150001, China; aria@hrbeu.edu.cn

**Keywords:** basalt-fiber-reinforced polymer (BFRP), split Hopkinson pressure bar (SHPB), strain rate effect, low-velocity impact

## Abstract

Basalt-fiber-reinforced polymer (BFRP) composites, utilizing a natural high-performance inorganic fiber, exhibit excellent weathering resistance, including tolerance to high and low temperatures, salt fog, and acid/alkali corrosion. They also possess superior mechanical properties such as high strength and modulus, making them widely applicable in aerospace and shipbuilding. This study experimentally investigated the mechanical properties of BFRP plates under various strain rates (10^−4^ s^−1^ to 10^3^ s^−1^) and directions using an electronic universal testing machine and a split Hopkinson pressure bar (SHPB).The results demonstrate significant strain rate dependency and pronounced anisotropy. Based on experimental data, relationships linking the strength of BFRP composites in different directions to strain rate were established. These relationships effectively predict mechanical properties within the tested strain rate range, providing reliable data for numerical simulations and valuable support for structural design and engineering applications. The developed strain rate relationships were successfully validated through finite element simulations of low-velocity impact.

## 1. Introduction

Basalt-fiber-reinforced polymer (BFRP) composites, incorporating a natural high-performance inorganic fiber, exhibit excellent weathering resistance (e.g., to temperature extremes, salt fog, and acid/alkali corrosion) and superior mechanical properties (high strength and modulus) [1,2]. Consequently, they find extensive use in demanding fields such as aerospace and shipbuilding. However, engineering structures may experience high-strain-rate loading during service. Furthermore, structural components made from fiber-reinforced polymer (FRP) composites, including BFRP composites, exhibit strain rate sensitivity under dynamic loading. Accurately assessing the load-bearing capacity and impact resistance of FRP composites requires a comprehensive understanding of their strain rate effects [3].

Researchers have employed various experimental techniques, including Split Hopkinson Pressure/Tensile Bars (SHPB/SHTB), Charpy impact, drop hammer tests, and high-velocity universal testing machines, to investigate the dynamic mechanical response of composites across different strain rates. These studies have established numerous experimental methods and standards related to strain-rate characterization [4].

For instance, Caverzan and Cadoni [5] used an improved SHPB to study fiber-reinforced concrete under low and high strain rates (150 s^−1^ to 300 s^−1^), finding that compressive stress increases with strain rate. Al-Mosawe et al. [6] investigated the dynamic tensile response of carbon fiber-reinforced polymer (CFRP) plates over strain rates from 10^−4^ s^−1^ to 100 s^−1^, reporting increases of 55% in tensile stress, 20% in elastic modulus, and 36% in ultimate strain compared to quasi-static conditions. Eskandari et al. [7] incorporated SHPB-derived strain-rate parameters into an ABAQUS VUMAT subroutine to analyze the viscoplastic behavior of composite laminates at low-to-moderate strain rates, confirming strength increases with strain rate and accurately capturing nonlinear behavior. Kim et al. [8] used Digital Image Correlation (DIC) to study polypropylene (PP) composites at a range of strain rates (0–100 s^−1^), observing increased tensile strength with higher rates. Li et al. [9] investigated 3D6d woven carbon fiber composites under high-strain-rate impact, demonstrating significant strain rate sensitivity that was influenced by braiding angle. Vieille et al. [10] proposed a strain-rate-dependent model for CF/PPS laminates using finite elements and an improved Norton-type viscoplastic model, focusing on matrix-dominated viscoelastic/viscoplastic effects.

While many fibers themselves exhibit negligible strain-rate effects, the polymer matrix in FRP composites significantly influences the composite’s overall strain rate sensitivity. Abdul-Latif et al. [11] developed an Abaqus user material subroutine accounting for strain-rate effects, reducing the error in maximum impact force simulation (at 5 m/s) from 35% to 15% compared to experiments. Zhang et al. [12] performed quasi-static and dynamic tensile tests on fiber/epoxy composites, finding significant increases in modulus and strength above 50 s^−1^, alongside changing failure modes, and derived predictive empirical formulas. Xin Shihong [13] numerically investigated the anti-penetration performance of FRP composites, proposing a dynamic progressive damage model implemented in Abaqus (6.14)/VUMAT. The Dynamic Increase Factor (DIF), defined as the ratio of dynamic to quasi-static strength or modulus [14], typically describes the strain-rate effect. Often, the DIF is assumed to be direction-independent. However, Gama et al. [15] found significant directional differences in strain-rate effects on modulus and strength, suggesting direction-specific DIF parameters improve prediction accuracy. Long et al. [16] proposed a nonlinear constitutive model for FRP composites under dynamic loads, capturing nonlinear transverse compression and in-plane shear behavior. Implemented in Abaqus via a user subroutine and validated through uniaxial tests and bird strike simulations, this model outperformed traditional ones in predicting dynamic response and failure modes. Furthermore, the Hashin failure criterion has demonstrated excellent performance for modeling composites under high-strain-rate conditions [17,18,19,20,21].

In summary, optimizing the design and application of composite structures necessitates investigating their strain-rate-dependent mechanical behavior. Therefore, experimental research on BFRP composites across multiple strain rates holds significant engineering value. This study experimentally examines the mechanical properties of BFRP plates in different directions under varying strain rates. Relationships linking the directional strength of BFRP composites to strain rate are established. These relationships effectively predict mechanical properties across the tested strain rate range (10^−4^ s^−1^ to 10^3^ s^−1^), providing reliable data for numerical simulations and supporting structural design and engineering applications under various loading conditions.

## 2. Materials and Methods

### 2.1. Materials

The basalt-fiber material used in this study was purchased from Anqing Kafu New Materials Technology Co., Ltd. (Anqing, China), with the model number X300P, which is an orthogonally woven plain fabric. The resin material selected was the No. 411 epoxy vinyl ester resin from Harbin MingRen Composite Materials Co., Ltd. (Harbin, China). This type of resin exhibits good wettability with basalt-fiber fabric, can be cured at room temperature, and possesses excellent elongation at break and impact toughness after curing.

### 2.2. Dynamic Mechanical Properties Testing of BFRP Composites

#### 2.2.1. Tensile Mechanical Properties Test

(1) In-Plane Tensile Properties

**Figure 1:**
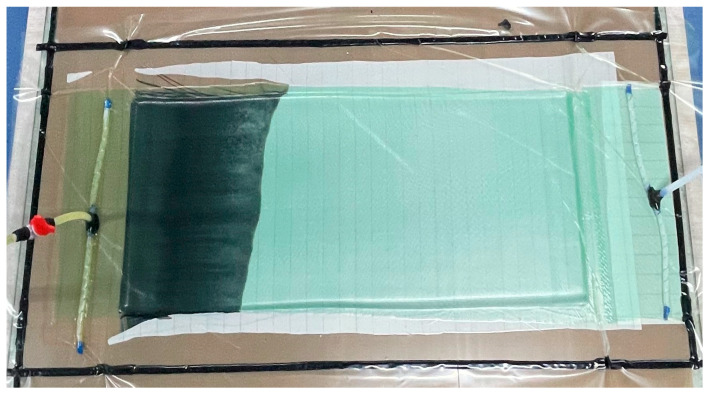
Preparation diagram of basalt-fiber laminates.

**Figure 2:**
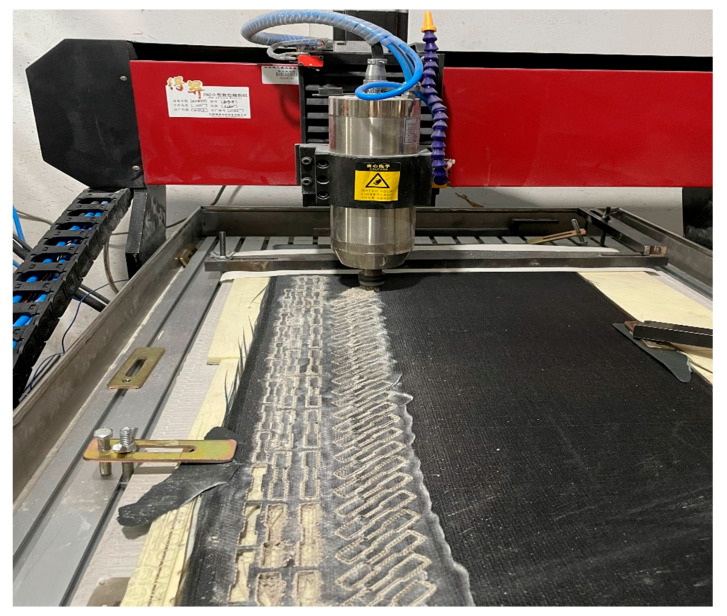
Basalt-fiber laminate tensile specimen engraving processing diagram.

**Figure 3:**
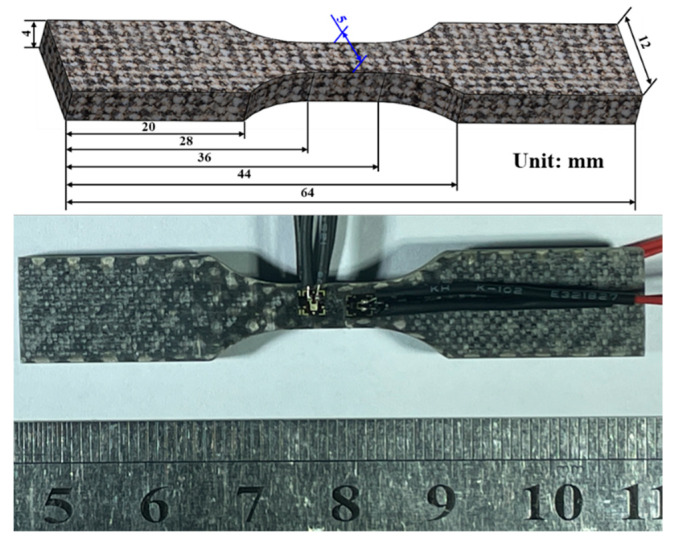
Basalt-fiber laminate tensile specimen.

**Figure 4:**
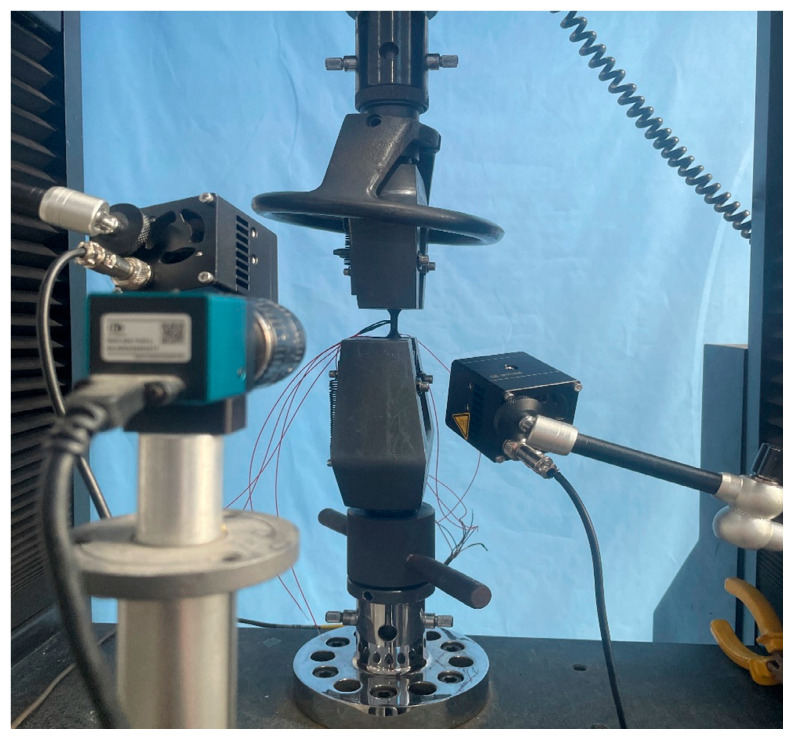
Picture of basalt-fiber laminate tensile test.

BFRP plates (4 mm thick, 16 layers of X300P fabric) were fabricated using Vacuum-Assisted Resin Transfer Molding (VARTM), as shown in Figure 1. Tensile specimens were prepared according to ASTM D3039 [22] and ASTM D3518 [23]. Due to the orthotropic nature of the orthogonal fabric, the in-plane X and Y directions exhibit identical properties; thus, testing focused only on the X direction. Specimens were cut from the same plate to minimize variability and edges were smoothed with 1500-grit sandpaper, as shown in Figure 2. Strain gauges with a resistance of 120 Ω were bonded in a half-bridge configuration along the X and Y directions, as shown in Figure 3. Tensile tests were performed on an INSTRON 5500R universal testing machine (INSTRON, Norwood, MA, USA) at crosshead displacement rates of 0.05, 0.5, 5, and 50 mm/min, corresponding to average strain rates of 10^−4^, 10^−3^, 10^−2^, and 10^−1^ s^−1^. Five replicates were tested per rate. Strain data were acquired using a Donghua DH8302 system (Jiangsu Donghua Test Technology Co., Ltd., Jingjiang, China) (50 Hz sampling), synchronized with the load cell. The test setup is shown in Figure 4.

(2) Out-of-Plane (Z-direction) Tensile Properties

**Figure 5:**
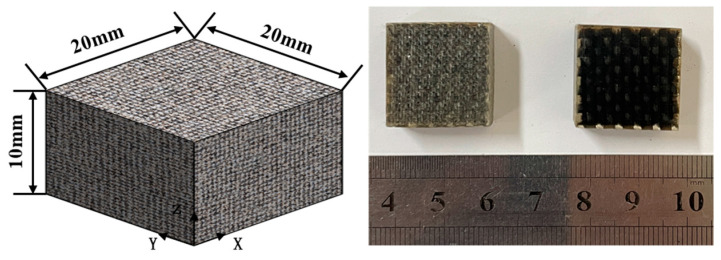
Basalt-fiber laminate out-of-plane tensile specimen.

**Figure 6:**
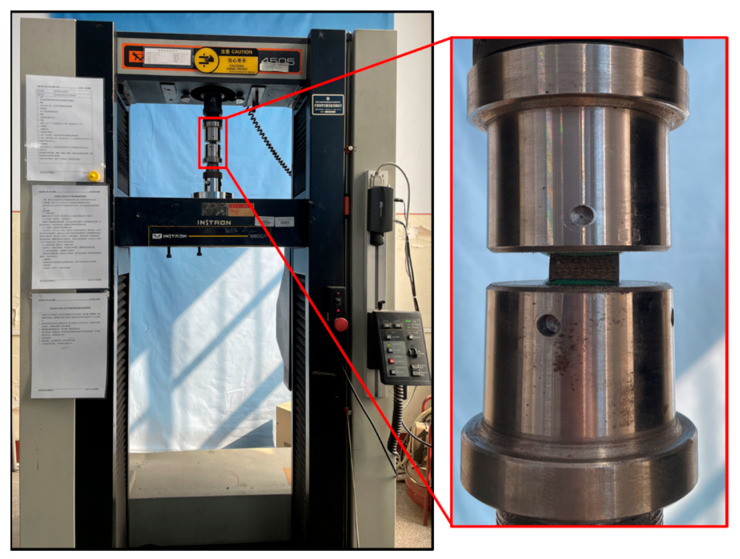
Picture of basalt-fiber laminate out-of-plane tensile test.

Plates for Z-direction testing (10 mm thick, 40 layers) were fabricated similarly. Specimens (20 mm × 20 mm × 10 mm, as shown in Figure 5) were machined per ASTM D7291/D7291M-15 [24] and bonded to fixtures using J-series composite adhesive film (Heilongjiang Academy of Petroleum Chemistry, Harbin, China). Tests used the INSTRON 5500R at displacement rates of 0.06, 0.6, 6, and 60 mm/min (average strain rates: 10^−4^, 10^−3^, 10^−2^, 10^−1^ s^−1^), with five replicates per rate. The setup is shown in Figure 6.

#### 2.2.2. Compressive Mechanical Properties Testing

**Figure 7:**
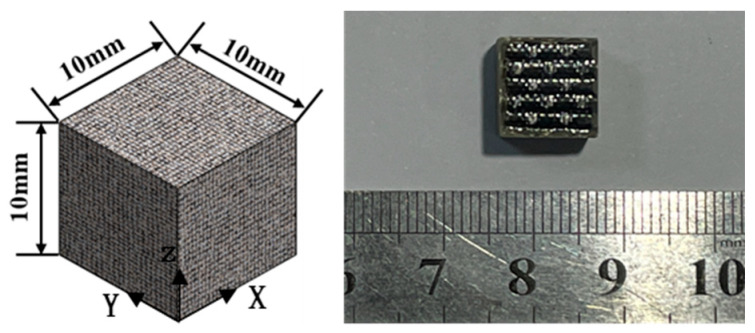
Basalt-fiber laminate compression specimen.

**Figure 8:**
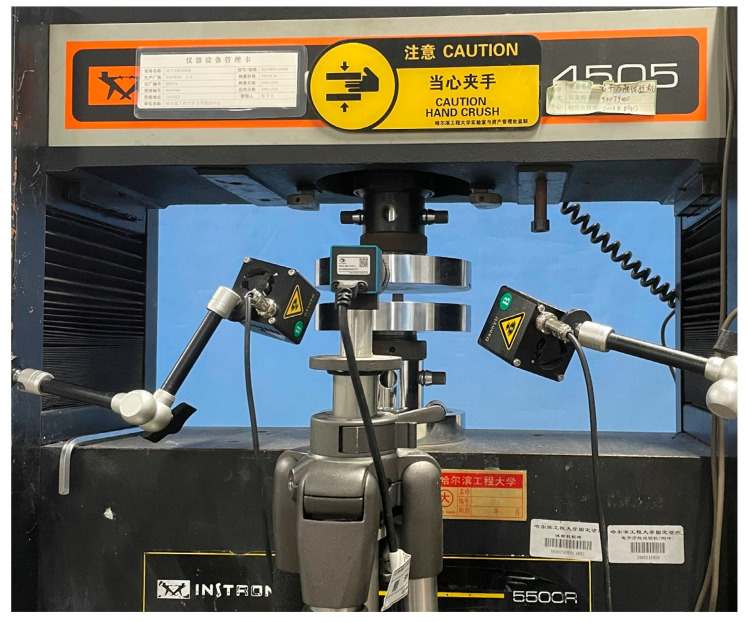
Picture of basalt-fiber laminate compression test.

Compression specimens were cut from plates identical to those used for Z-direction tension (10 mm thick, 40 layers) using a CNC machine (Shenzhen Songpu Industrial Group Co., Ltd., Shenzhen, China), with edges smoothed, as shown in Figure 7. Low-strain-rate compression tests (X and Z directions) used the INSTRON 5500R ((INSTRON, Norwood, MA, USA)) at displacement rates of 0.06, 0.6, 6, and 60 mm/min (average strain rates: 10^−4^, 10^−3^, 10^−1^, 10^−1^ s^−1^), with five replicates per rate/direction, as illustrated in Figure 8. Medium-to-high strain rate compression tests (X and Z directions) employed a 20-mm diameter SHPB setup (bars made of 60Si2Mn spring steel; impact bar: 300 mm, incident bar: 2500 mm, transmitted bar: 2000 mm, absorber bar: 1000 mm).

#### 2.2.3. Shear Mechanical Properties Testing

(1) In-Plane Shear Properties

The in-plane shear strength of basalt-fiber materials was tested according to ASTM D3518 [23]. The shear test specimens were identical in size to the tensile specimens and were cut at ±45° to the fiber direction using a CNC engraving machine (Shenzhen Songpu Industrial Group Co., Ltd., Shenzhen, China). The testing conditions and data acquisition equipment were consistent with those used in the tensile tests.

(2) Out-of-Plane Shear Properties

**Figure 9:**
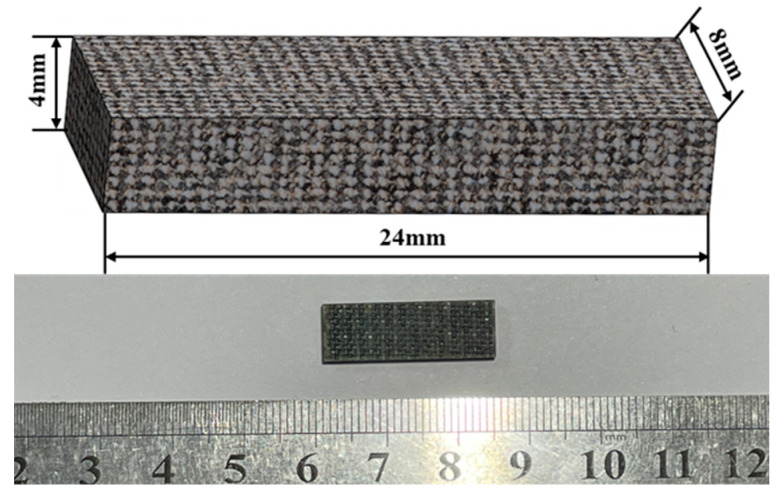
Basalt-fiber laminate short-beam shear specimen.

**Figure 10:**
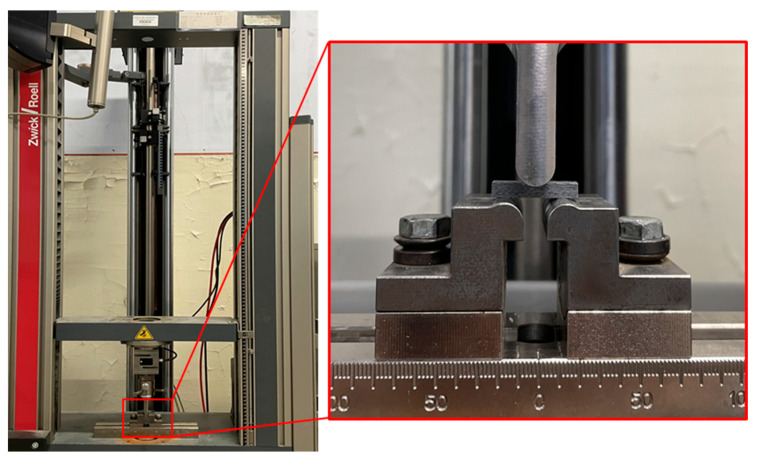
Picture of basalt-fiber laminate short-beam shear test.

Out-of-plane shear strength was tested according to ASTM D 2344 [25], using a short-beam shear test. The short-beam specimens were cut from the same basalt-fiber laminate using a CNC engraving machine, as shown in Figure 9. The tests were conducted using a Ziwick Z010 universal testing machine (Ziwick, Ulm, Germany) with a maximum load capacity of 10 kN. The crosshead displacement rates were set at 0.024 mm/min, 0.24 mm/min, 2.4 mm/min, 24 mm/min, and 240 mm/min, corresponding to average strain rates of 10^−4^ s^−1^, 10^−3^ s^−1^, 10^−2^ s^−1^, 10^−1^ s^−1^, and 10^0^ s^−1^, respectively. Five replicate tests were conducted for each strain rate. The experimental setup for the short beam shear test is shown in Figure 10.

### 2.3. Validation of the Strain Rate Effect in a Low-Velocity Impact Simulation

A Fortran VUMAT subroutine incorporating the 3D Hashin failure criterion and the directional strain rate relationships established in this study was developed for implementation in ABAQUS (2022)/Explicit.

**Figure 11:**
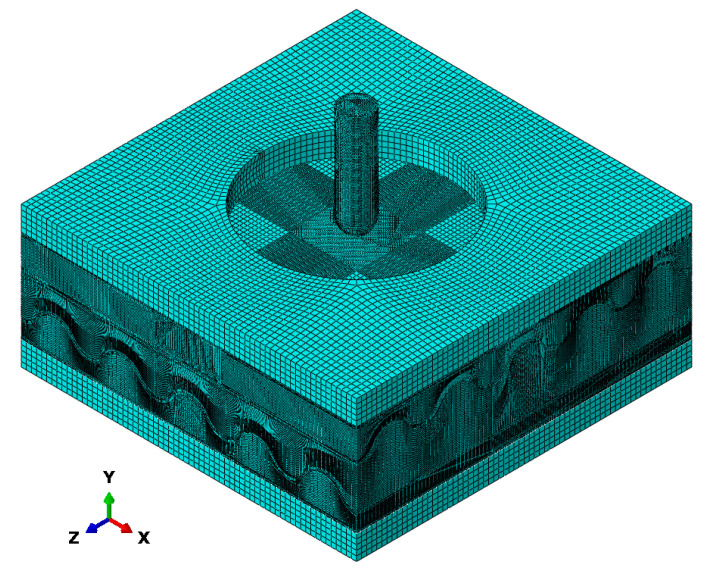
Low-velocity drop hammer impact for the finite element model mesh.

A 3D finite element model replicating the H-CS-T-160J experimental conditions described in [26] was built, as shown in Figure 11. The sandwich structure was modeled integrally, with the fiber layers and foam core connected via shared nodes. The influence of incorporating strain-rate effects on simulation accuracy was assessed by comparing the results with experimental data.

**Figure 12:**
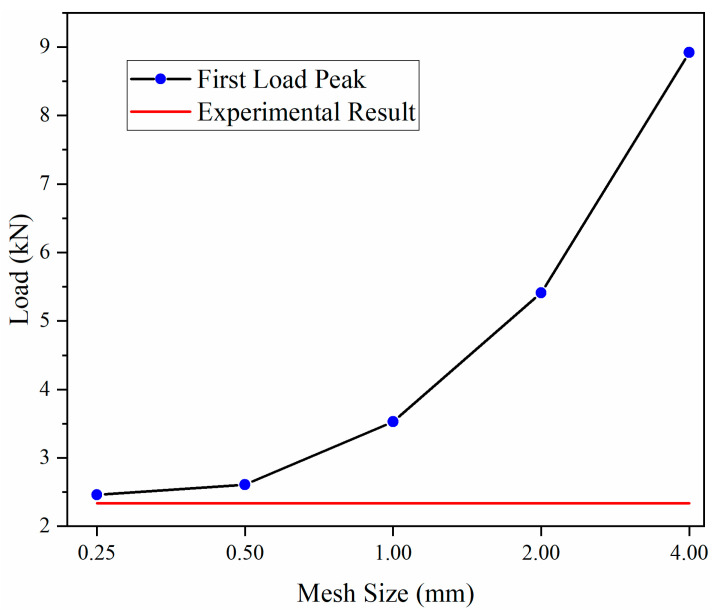
Correlation between mesh size and the numerical simulation results.

To investigate the influence of mesh size on computational accuracy, finite element models of the corrugated sandwich structure (with a corrugation arc radius of 8 mm) were established with mesh sizes of 4 mm, 2 mm, 1 mm, 0.5 mm, and 0.25 mm in the impact zone. To optimize computational efficiency, the first load peak was selected as the evaluation criterion, with experimental data serving as the reference. The relationship between mesh size and computational results is shown in Figure 12. As illustrated in Figure 12, the computational results progressively converge toward the experimental values with decreasing mesh size. By considering the optimal balance between computational accuracy and efficiency, a mesh size of 0.5 mm was adopted for the impact zone in this study (Supplementary Materials).

## 3. Results

### 3.1. In-Plane Tensile Strength

The ultimate tensile strength of the basalt-fiber material was calculated using Equation (1), with results rounded to two decimal places. The tensile stress at each data point was determined using Equation (2).

(1) $$F^{tu}=P^{\max}/A$$

(2) $$\sigma_{i}=P_{i}/A$$

where:

$F^{tu}$ is the ultimate tensile strength, in MPa;

$P^{\max}$ is the maximum load before failure, in N;

$P_{i}$ is the load at the *i*th data point, in N; 

A is the average effective cross-sectional area of the specimen, in mm^2^.

**Figure A1:**
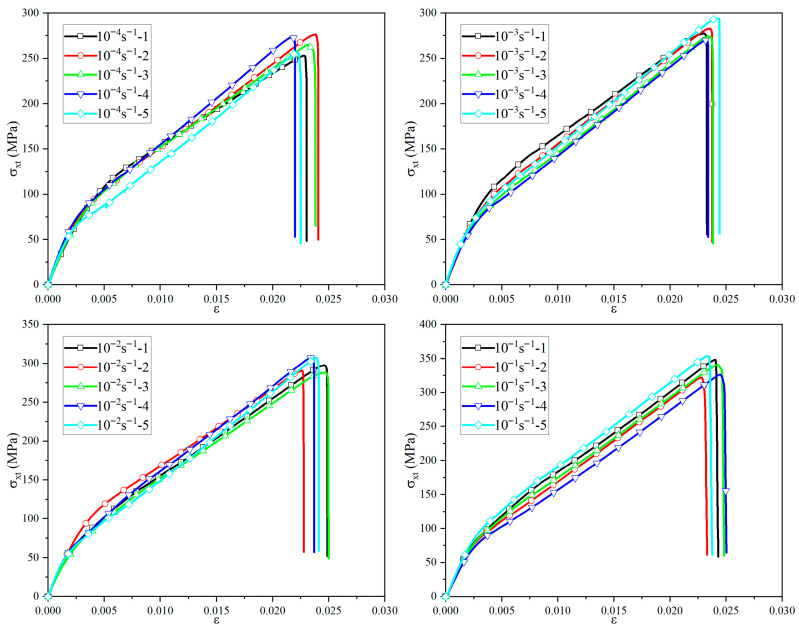
In-plane tensile stress–strain relationship curves of basalt-fiber laminates at different strain rates.

The tensile stress–strain curves of the basalt-fiber laminate under different strain rates were obtained using Equation (2). Five repeated tests were conducted for each strain rate, as shown in Figure A1. The tensile strength of the material under different strain rates was calculated using Equation (1), and the standard deviation of the results was calculated for each strain rate. The experimental results are shown in Table 1.

### 3.2. Out-of-Plane Tensile Strength

The out-of-plane ultimate tensile strength of the basalt-fiber material was similarly calculated using Equation (1), with results rounded to two decimal places. The tensile stress at each data point was determined using Equation (2).

**Figure A2:**
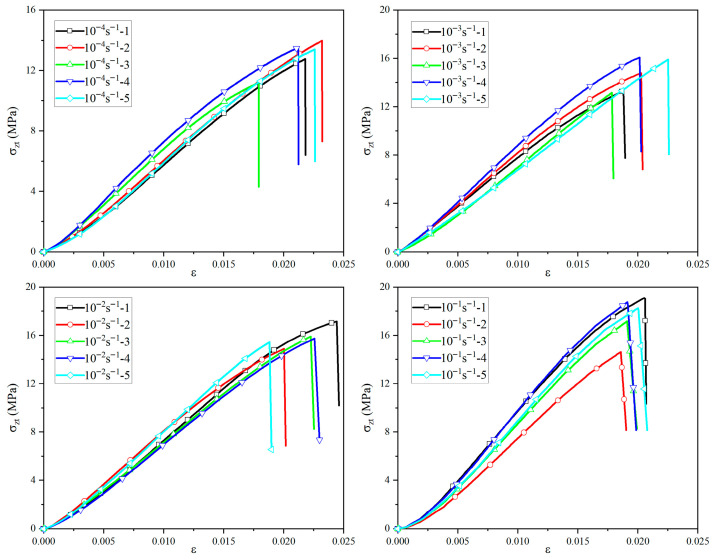
Out-of-plane tensile stress–strain relationship curves of basalt-fiber laminates at different strain rates.

Using Equation (2), the out-of-plane tensile stress–strain curves of the basalt-fiber laminate under different strain rates were obtained. Five repeated tests were conducted for each strain rate, as shown in Figure A2. By combining the results with Equation (1), the out-of-plane tensile strength of the material under different strain rates was calculated, and the standard deviation of the results was calculated for each strain rate. The experimental results are shown in Table 2.

### 3.3. In-Plane Compressive Strength

**Figure A3:**
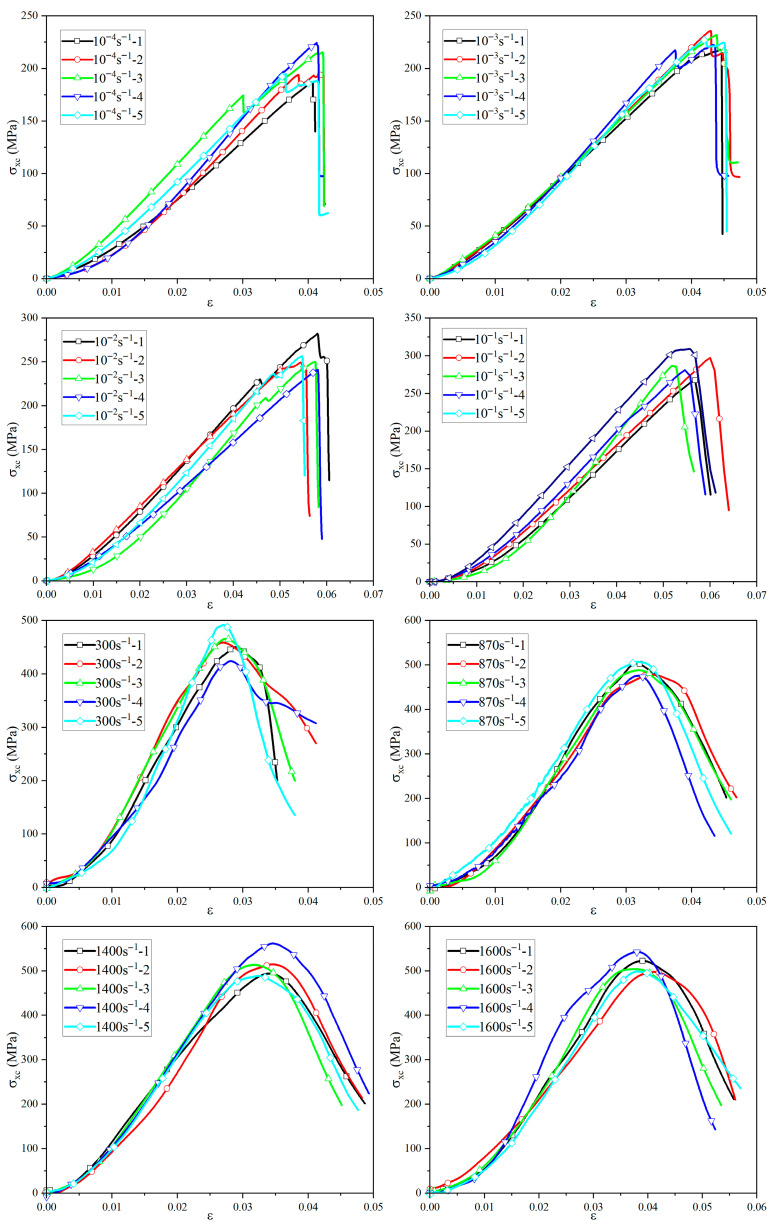
In-plane compressive stress–strain curves of basalt-fiber laminates at different strain rates.

**Figure A4:**
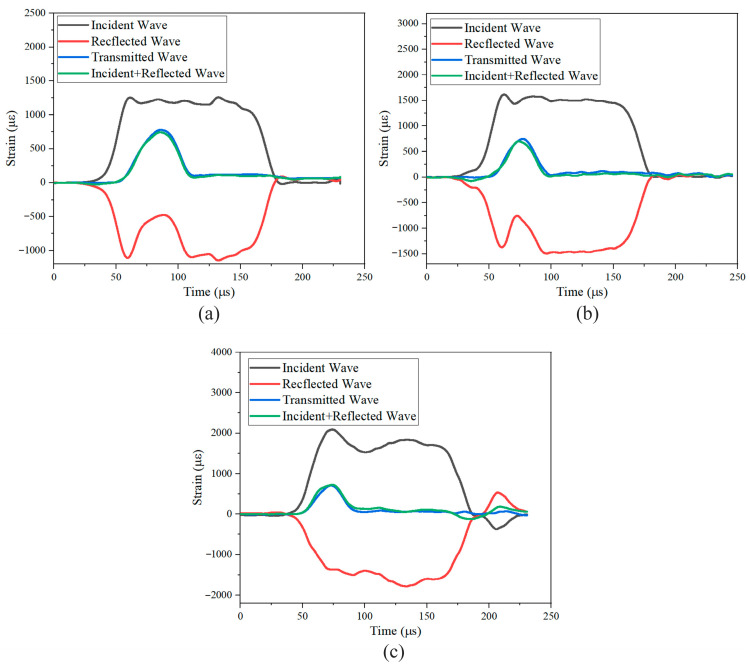
The experimental waveforms and stress balance conditions of basalt-fiber-laminated plates at different strain rates: (**a**) 870 s^−1^, (**b**) 1400 s^−1^, and (**c**) 1600 s^−1^.

The stress–strain curves of the basalt-fiber laminate under in-plane compression were obtained, using Equation (2), for strain rates ranging from 10^−4^ s^−1^ to 10^−1^ s^−1^, with five repeated tests for each strain rate. For strain rates from 300 s^−1^ to 1600 s^−1^, the stress–strain curves were obtained using the three-wave method and Equation (4), again with five repeated tests for each strain rate. As depicted in Figure A3, the in-plane compressive strength of the material across eight strain rates from 10^−4^ s^−1^ to 1600 s was calculated using Equations (2)–(4). The standard deviation of the results was also computed for each strain rate. The experimental results are presented in Table 3. The incident, reflected, and transmitted waveforms under varying strain rates were processed and analyzed, as shown in Figure A4. The results demonstrate that the tested specimen maintained satisfactory stress equilibrium during dynamic loading, thereby validating the reliability of the SHPB experimental methodology.

(3) $$F^{cu}=P^{\max}/A$$

where:

$F^{cu}$ is the ultimate compressive strength, in MPa;

$P^{\max}$ is the maximum load before failure, in N.

(4) $$\left\{\begin{array}{l} \dot{\varepsilon }(t)=\frac{1}{l_{s}}(v_{1}-v_{2})=\frac{c}{l_{s}}(\varepsilon_{i}-\varepsilon_{r}-\varepsilon_{t})\\ \varepsilon (t)=\frac{c}{l_{s}}\int_{0}^{t}(\varepsilon_{i}-\varepsilon_{r}-\varepsilon_{t})dt\\ \sigma (t)=\frac{1}{2}\left[\frac{A}{A_{s}}E(\varepsilon_{i}+\varepsilon_{r})+\frac{A}{A_{s}}E\varepsilon_{t}\right]=\frac{A}{2A_{s}}E(\varepsilon_{i}+\varepsilon_{r}+\varepsilon_{t}) \end{array}\right.$$

where:

c, E, A are the elastic wave velocity, elastic modulus, and cross-sectional area of the SHPB experimental device bar, respectively;

$l_{s}$, $A_{s}$ are the thickness and cross-sectional area of the specimen, respectively;

$\varepsilon_{i}$, $\varepsilon_{r}$, $\varepsilon_{t}$ are the incident wave strain, reflected wave strain, and transmitted wave strain collected in the SHPB experiment, respectively;

$v_{1}$, $v_{2}$ are the particle velocities at the front and rear ends of the specimen.

### 3.4. Out-of-Plane Compressive Strength

**Figure A5:**
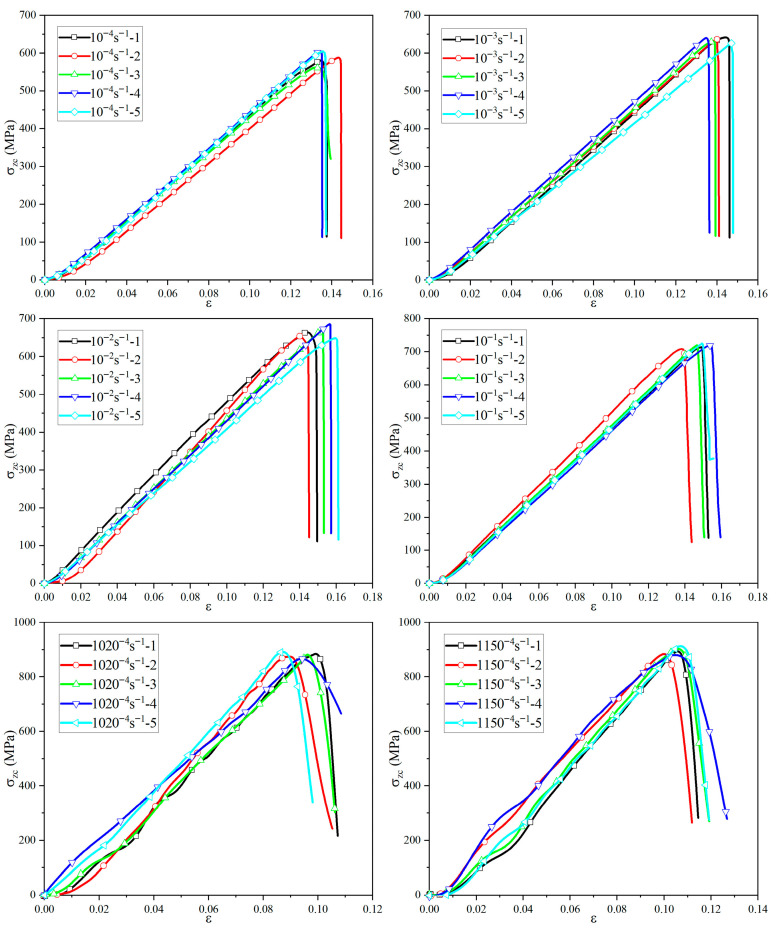
Out-of-plane compressive stress–strain relationship of basalt-fiber laminates at different strain rates.

Using Equations (2)–(4), the stress–strain curves for the out-of-plane compressive strength of the basalt-fiber material were obtained at various strain rates, as shown in Figure A5. The ultimate out-of-plane compressive strength of the material was extracted for six strain rates ranging from 10^−4^ s^−1^ to 1150 s^−1^. The standard deviation of the results was calculated for each strain rate. The experimental results are presented in Table 4.

**Figure A6:**
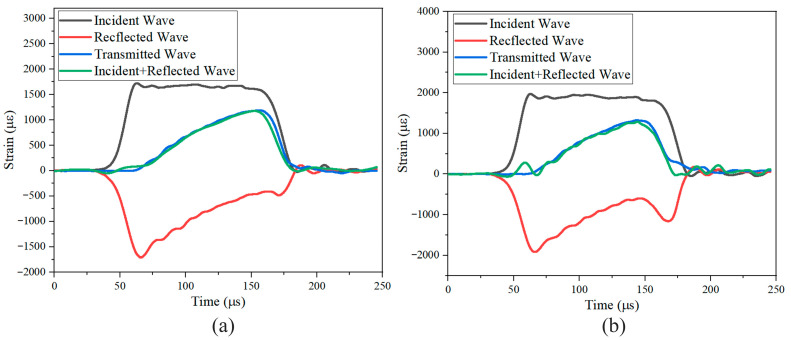
The experimental waveforms and stress balance conditions of basalt-fiber-laminated plates at different strain rates: (**a**) 1020 s^−1^ and (**b**) 1150 s^−1^.

Similarly, for the incident wave, reflected wave, and transmitted wave of the out-of-plane compression test under different strain rates, processing was carried out as shown in Figure A6. As can be seen from Figure A6, during the test, the tested specimen maintained satisfactory stress equilibrium, which proved the reliability of the SHPB test results.

### 3.5. In-Plane Shear Strength

**Figure A7:**
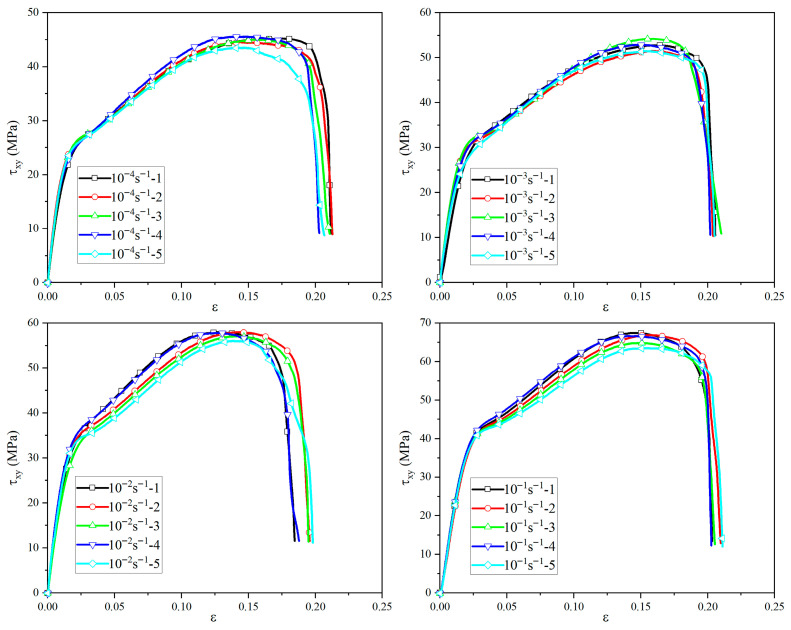
In-plane shear strength stress–strain relationship curves of basalt-fiber laminates at different strain rates.

During in-plane shear testing, if the specimen does not eventually fail when the shear strain hits 5%, the shear strain data beyond 5% were deleted in data processing. The stress corresponding to the 5% shear strain was deemed the maximum shear stress for recording and calculation purposes. The shear stress at each data point was determined using Equation (5), and stress–strain curves for different strain rates was be plotted, as shown in Figure A7. The in-plane shear strength of the material at 5% shear strain under different strain rates was extracted. Specifically, the in-plane shear strength under four strain rates from 10^−4^ s^−1^ to 10^−1^ s^−1^ was obtained, along with the standard deviation of results for each strain rate. The experimental results are presented in Table 5.

(5) $$\tau_{12i}=\frac{p_{i}}{2A}$$

where:

$\tau_{12i}$ is the shear stress at the *i*th data point, in MPa;

$p_{i}$ is the load at the *i*th data point, in N;

A is the average effective cross-sectional area of the specimen, in mm^2^.

### 3.6. Out-of-Plane Shear Strength

**Figure A8:**
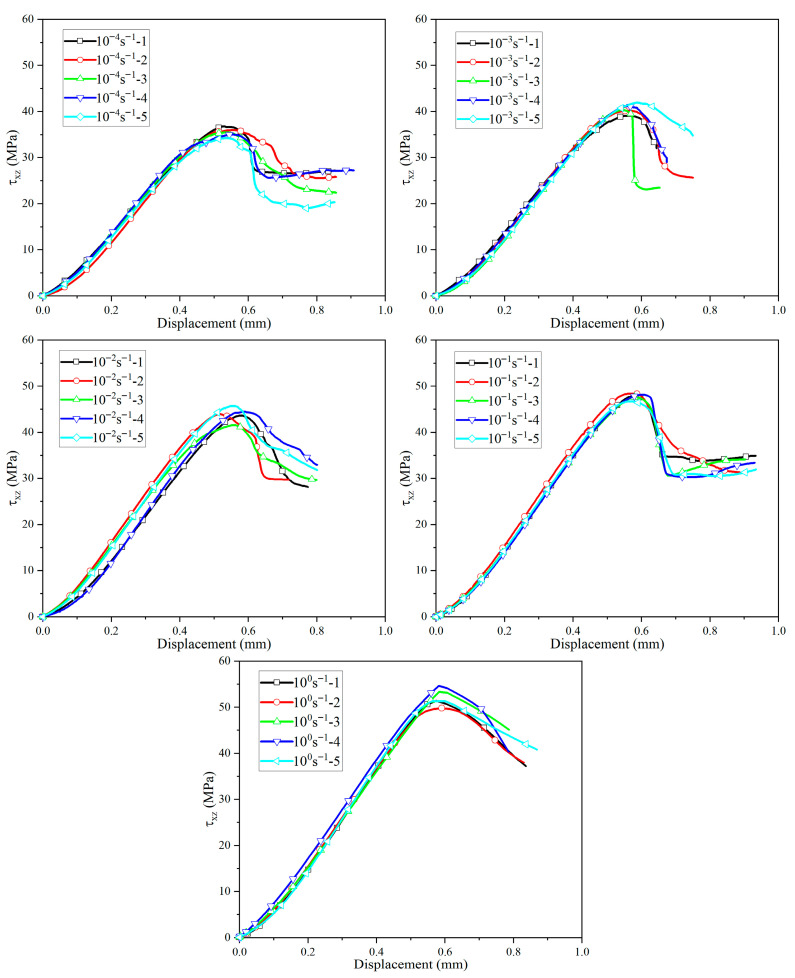
Out-of-plane shear strength stress-displacement relationship curves of basalt-fiber laminates at different strain rates.

In the out-of-plane shear experiment, the short-beam shear strength can be determined using Equation (6). This equation also allows for the calculation of short-beam shear strength corresponding to different loads, enabling the plotting of the relationship curve between compressive displacement and short-beam strength under various strain rates, as shown in Figure A8. By extracting the short-beam shear strength values of the material under different strain rates, the out-of-plane shear strength of the material under five strain rates from 10^−4^ s^−1^ to 100 s^−1^ can be obtained. The standard deviation of the results was calculated for each strain rate. The experimental results are presented in Table 6.

(6) $$F^{sbs}=0.75\times \frac{P_{m}}{b\times h}$$

where:

$F^{sbs}$ is the short beam strength, in MPa;

$P_{m}$ is the maximum load in the experiment, in N;

b is the width of the specimen, in mm;

h is the thickness of the specimen, in mm.

### 3.7. The Strain Rate Effect of Basalt-Fiber Composite Materials

To more precisely characterize the relationship between the strength of materials and the strain rate, a power–law relationship formula was established [27].

(7) $$\frac{\sigma \left(\varepsilon \right)}{\sigma_{0}\left(\varepsilon \right)}={\left(\frac{\dot{\varepsilon }}{{\dot{\varepsilon }}_{0}}\right)}^{m}$$

where ${\dot{\varepsilon }}_{0}$ is the reference strain rate, $\dot{\varepsilon }$ is the stress under the reference strain rate, and m is the power index.

**Figure 13:**
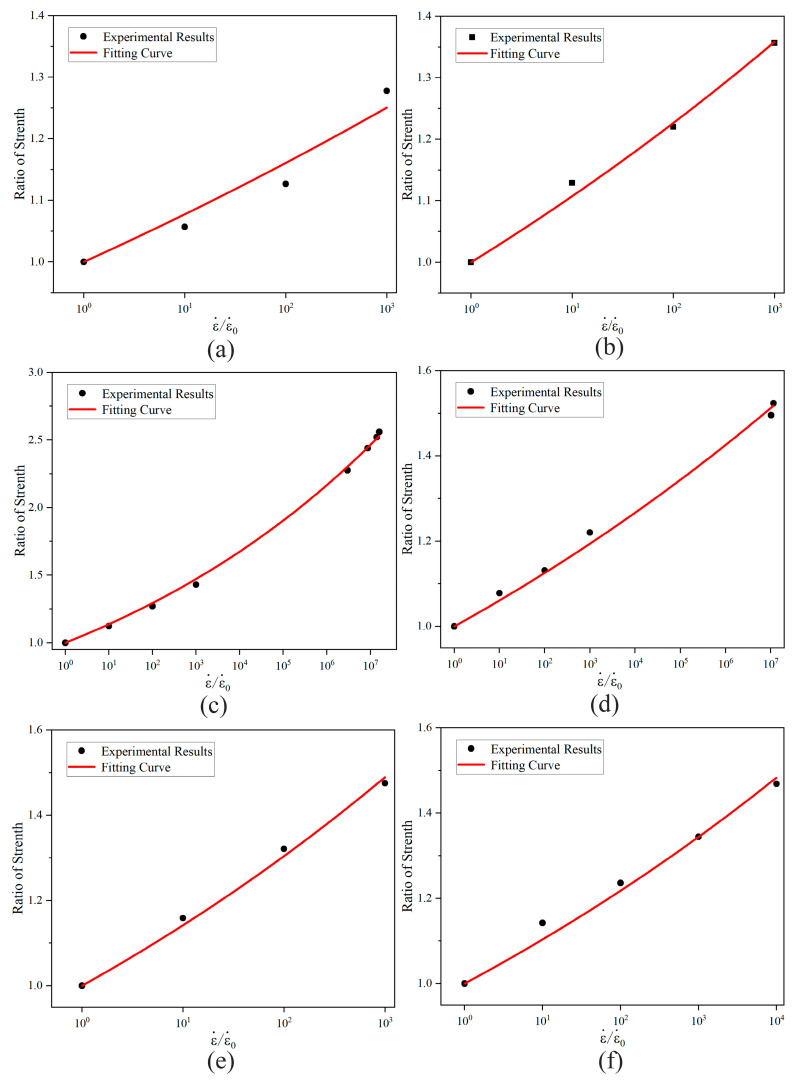
Effect of strain rate on strength values of basalt-fiber laminates in different directions: (**a**) in-plane tensile strength, (**b**) out-of-plane tensile strength, (**c**) in-plane compressive strength, (**d**) out-of-plane compressive strength, (**e**) in-plane shear strength, and (**f**) out-of-plane shear strength.

The mechanical parameters of basalt-fiber materials at a reference strain rate of 10^−4^ s^−1^ are presented in Table 7. The average strength ratio and strain-rate sensitivity ratio of basalt fibers in each direction were nonlinearly fitted using Equation (7). Additionally, scatter plots and corresponding nonlinear fitting curves for the strength ratio and strain rate sensitivity ratio of basalt fibers in each direction were generated, as shown in Figure 13.

**Figure 14:**
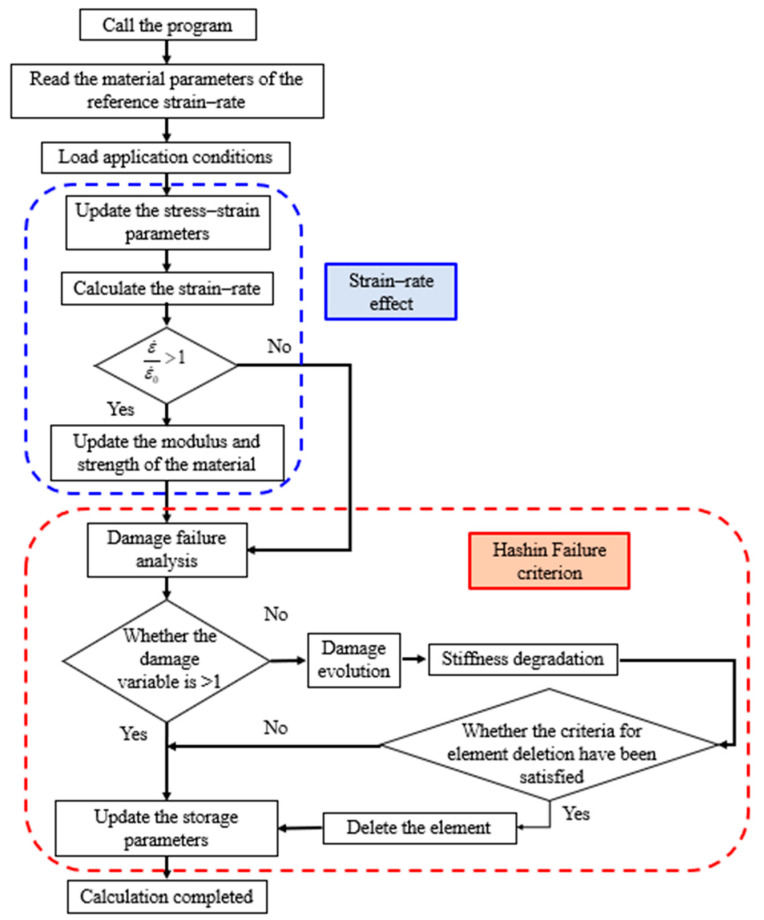
Simulation process of the calculation model based on the Hashin failure criterion considering the strain-rate effect of the materials.

From Figure 13, it can be observed that the strain rate effect power index for tensile strength in the in-plane X direction of the fiber is 0.03238, while in the out-of-plane Z direction, it is 0.04433. For compressive strength, the strain rate effect power index in the in-plane X direction is 0.05594, and in the out-of-plane Z direction, it is 0.02566. For shear strength, the strain rate effect power index in the in-plane direction is 0.05765, and in the out-of-plane direction, it is 0.0428. These results indicate that the strain-rate effects on the strength values of basalt-fiber composites differ across directions, all exhibiting a strong nonlinear growth trend. The simulation process of the calculation model based on the Hashin failure criterion, considering the strain rate effect of the materials, is shown in Figure 14.

## 4. Discussion

**Figure 15:**
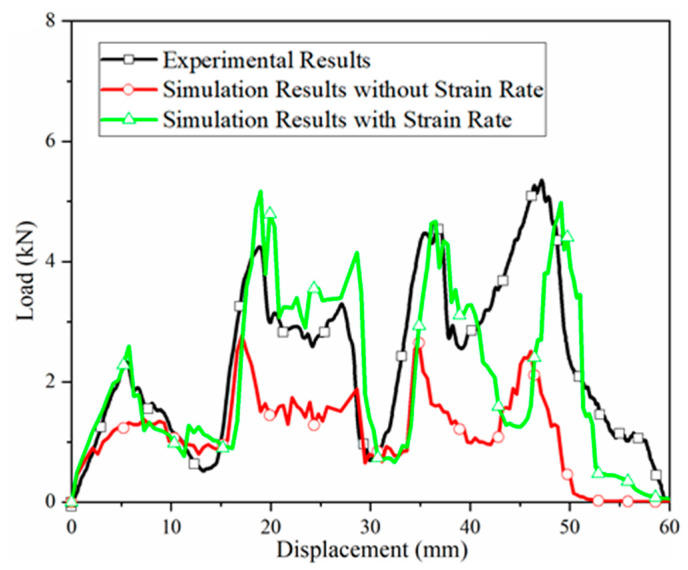
Contact force history of the hemisphere hammer head impacting the trough position of the orthogonal corrugated sandwich structure.

The reaction force between the hemispherical impactor and the sandwich structure, along with impactor displacement, were extracted from simulations both with and without material-strain-rate effects. Force-displacement curves were plotted and compared with experimental data, as shown in Figure 15. The simulation methodology proved feasible, as both cases produced curves with consistent trends. However, neglecting the strain-rate effect resulted in a force-displacement curve deviating significantly from the experiments. In contrast, incorporating the strain-rate effect yielded excellent agreement with the experimental data, underscoring its critical importance under dynamic loading.

Four distinct load peaks, identified in Figure 15, were compared between simulations and experiments, as presented in Table 8. Neglecting strain-rate effects led to large errors: the minimum error (peak 2) was 34.59% and the average error across peaks was 42.49%. These errors are unacceptable for predictive modeling. Incorporating the strain-rate effect substantially reduced errors: the minimum error (peak 3) was 3.79% and the average error was 10.87%, representing a 31.62% reduction. This validates the accuracy of the developed BFRP strain-rate relationships and highlights the necessity of accounting for strain-rate effects in low-velocity impact simulations.

**Figure 16:**
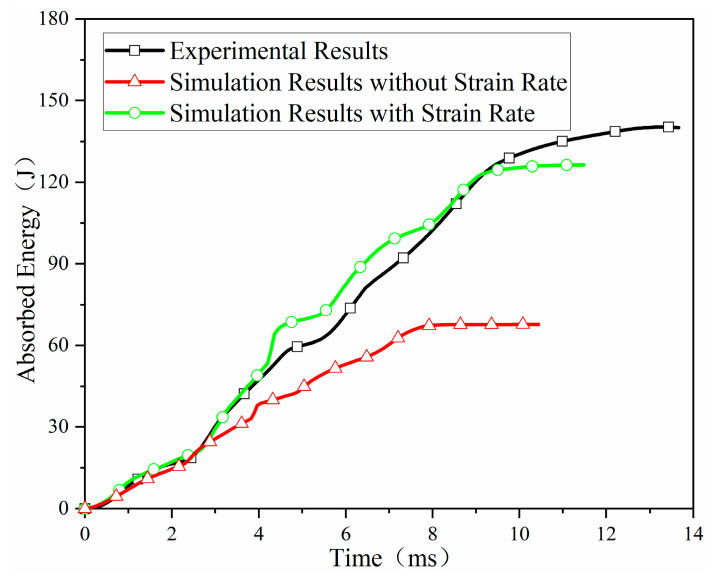
Energy absorption history of the hemisphere hammer head impacting the trough position of the orthogonal corrugated sandwich structure.

Energy absorption over time for the sandwich structure was extracted from the simulations and compared to the experimental results, as shown in Figure 16. Experimentally, total energy absorption under a 160 J impact was 140.58 J. The simulation neglecting strain-rate effects predicted 67.67 J (51.86% error). The simulation incorporating strain-rate effects predicted 126.44 J (10.06% error), representing a 41.80% reduction in error. Furthermore, the energy absorption history considering strain-rate effects closely matched the experimental curve, unlike the case neglecting it.

From the above research results, it can be known that when conducting low-velocity impact numerical simulation, the strain-rate effect of the material cannot be ignored. This also indicates that the composite material calculation VUMAT subroutine proposed in this study, which considers the material strain-rate effect based on the Hashin failure criterion, is effective and reliable in low-velocity impact simulation conditions.

However, although using a single power–law model to describe the nonlinear relationship between strain rate and strength under different loading conditions is a practical approach [27,28,29,30], this model mainly focuses on expressing the macroscopic mechanical behavior of the material and fails to clearly define the dominant roles of the matrix and fibers in the contribution to the strain-rate effect and failure behavior within the composite material from a mechanistic perspective. This may have certain impacts on the calculation accuracy. Combining the above model with molecular dynamics models or multi-scale calculation models might be an effective method to explore its mechanism.

## 5. Conclusions

This study experimentally investigated the strain rate dependency of BFRF composites using universal testing machines and SHPB. A VUMAT subroutine incorporating a three-dimensional Hashin failure criterion with strain rate effects was developed. The numerical simulations of low-velocity impacts on basalt-fiber structures were performed under identical conditions to the experiments, and the results were compared. The key conclusions are as follows:

(1) BFRP composites exhibit significant strain-rate dependency, with strength increasing as strain rate increases.
(2) The strain-rate effect is directionally dependent, showing varying sensitivity across different material orientations.
(3) The relationship between strength and strain rate in different directions follows a nonlinear trend, well-described by the established power–law relationships.
(4) The developed strain-rate relationships for BFRP composites were successfully validated in low-velocity impact simulations, demonstrating high accuracy compared to experimental results when incorporated into the numerical model.

## Figures and Tables

**Table 1:** In-plane tensile stress values and standard deviations of basalt-fiber laminates at different strain rates.

Strain Rate (s^−1^)	In-Plane Tensile Strength (MPa)					Average Tensile Strength (MPa)	Standard Deviation (MPa)
	Specimen 1	Specimen 2	Specimen 3	Specimen 4	Specimen 5		
10^−4^	273.42	276.50	264.86	256.32	253.14	264.85	10.24
10^−3^	294.33	282.95	277.42	274.58	270.23	279.90	9.29
10^−2^	307.32	307.48	297.71	291.10	288.41	298.40	8.88
10^−1^	353.74	348.44	341.17	322.24	326.35	338.39	13.69

**Table 2:** Out-of-plane tensile stress values and standard deviations of basalt-fiber laminates at different strain rates.

Strain Rate (s^−1^)	Out-of-Plane Tensile Strength (MPa)					Average Tensile Strength (MPa)	Standard Deviation (MPa)
	Specimen 1	Specimen 2	Specimen 3	Specimen 4	Specimen 5		
10^−4^	11.20	13.53	12.77	13.40	13.98	12.98	1.08
10^−3^	13.31	14.79	16.09	15.91	13.15	14.65	1.39
10^−2^	17.15	14.90	15.92	15.75	15.43	15.83	0.83
10^−1^	18.78	19.12	18.27	17.17	14.66	17.60	1.80

**Table 3:** In-plane compressive stress values and standard deviations of basalt-fiber laminates under different strain rates.

Strain Rate (s^−1^)	In-Plane Compressive Strength (MPa)					Average Compressive Strength (MPa)	Standard Deviation (MPa)
	Specimen 1	Specimen 2	Specimen 3	Specimen 4	Specimen 5		
10^−4^	187.07	224.23	214.90	186.85	193.93	201.39	17.15
10^−3^	235.63	231.70	224.42	222.27	217.93	226.39	7.18
10^−2^	281.74	257.07	249.93	249.50	241.37	255.92	15.47
10^−1^	309.18	297.24	285.48	267.40	281.05	288.07	15.91
300	466.23	459.06	450.14	424.56	492.40	458.48	24.65
870	505.15	488.27	481.60	476.89	506.45	491.67	13.52
1400	515.42	513.54	495.68	487.57	528.85	508.21	16.50
1600	522.63	520.20	497.07	498.78	542.67	516.27	18.89

**Table 4:** Out-of-plane compressive stress values and standard deviations of basalt-fiber laminates at different strain rates.

Strain Rate (s^−1^)	Out-of-Plane Compressive Strength (MPa)					Average Compressive Strength (MPa)	Standard Deviation (MPa)
	Specimen 1	Specimen 2	Specimen 3	Specimen 4	Specimen 5		
10^−4^	605.66	562.37	588.04	604.63	581.85	588.51	17.90
10^−3^	630.00	626.02	642.46	638.48	639.45	635.28	6.94
10^−2^	654.96	643.82	673.86	685.31	670.50	665.69	16.34
10^−1^	720.21	725.61	719.51	708.98	715.09	717.88	6.22
1020	885.30	870.41	884.25	865.57	894.41	879.99	11.78
1150	905.14	888.35	895.24	879.28	913.20	896.24	13.40

**Table 5:** In-plane shear strength stress values and standard deviations of basalt-fiber laminates at different strain rates.

Strain Rate (s^−1^)	In-Plane Shear Strength (MPa)					Average Shear Strength (MPa)	Standard Deviation (MPa)
	Specimen 1	Specimen 2	Specimen 3	Specimen 4	Specimen 5		
10^−4^	31.74	31.31	31.13	31.11	30.96	31.25	0.30
10^−3^	36.92	36.51	36.06	35.82	35.74	36.21	0.50
10^−2^	43.38	43.09	40.97	40.06	38.92	41.28	1.93
10^−1^	47.81	46.83	45.94	45.26	44.63	46.09	1.26

**Table 6:** Out-of-plane shear strength stress values and standard deviations of basalt-fiber laminates at different strain rates.

Strain Rate (s^−1^)	Out-of-Plane Shear Strength (MPa)					Average Shear Strength (MPa)	Standard Deviation (MPa)
	Specimen 1	Specimen 2	Specimen 3	Specimen 4	Specimen 5		
10^−4^	36.81	36.01	35.47	34.97	34.26	35.50	0.97
10^−3^	42.01	41.21	40.16	40.33	39.08	40.56	1.11
10^−2^	45.68	44.01	41.53	44.50	43.68	43.88	1.51
10^−1^	48.37	48.16	47.82	47.60	46.71	47.73	0.64
10^0^	54.63	53.40	51.47	51.32	49.85	52.13	1.88

**Table 7:** Material parameters of basalt-fiber laminates at the reference strain rate.

$E_{1}$ (GPa)	$E_{2}$ (GPa)	$E_{3}$ (GPa)	$G_{12}$ (GPa)	$G_{13}$ (GPa)	$G_{23}$ (GPa)	$\nu_{12}$	$\nu_{13}$	$\nu_{23}$	
25.83	25.83	9.00	23.809	4.339	4.339	0.28	0.148	0.148	
$\sigma_{XT}$ (MPa)	$\sigma_{XC}$ (MPa)	$\sigma_{YT}$ (MPa)	$\sigma_{YC}$ (MPa)	$\sigma_{ZT}$ (MPa)	$\sigma_{ZC}$ (MPa)	$\tau_{XY}$ (MPa)	$\tau_{XZ}$ (MPa)	$\tau_{YZ}$ (MPa)	$Z_{T}$ (MPa)
264.85	201.40	264.85	201.40	12.98	588.34	264.85	31.25	31.25	12.98

**Table 8:** Comparison of the numerical simulation results and experimental load peaks.

Load Peaks	Experimental Results (kN)	Simulation Results Without Strain Rates (kN)	Errors	Simulation Results with Strain Rates (kN)	Errors
First	2.34	1.38	41.03%	2.61	11.54%
Second	4.25	2.78	34.59%	5.16	21.41%
Third	4.49	2.63	41.43%	4.66	3.79%
Fourth	5.35	2.52	52.90%	4.99	6.73%

## Data Availability

The original contributions presented in the study are included in the article/Supplementary Material, further inquiries can be directed to the corresponding author.

## Supplementary Materials

The following supporting information can be downloaded at: https://www.mdpi.com/article/10.3390/ma18153637/s1, File S1: Failure criteria for fiber composite materials considering the effect of material strain rate.
