# Supplementary material for: Strain-Rate Effects on the Mechanical Behavior of Basalt-Fiber-Reinforced Polymer Composites: Experimental Investigation and Numerical Validation
Source: Materials (Basel). 2025 Aug 1;18(15):3637. doi: 10.3390/ma18153637 (PMC12348519; doi:10.3390/ma18153637)
Supplement: Supplementary file 1 [file materials-18-03637-s001.zip › materials-3737405-supplementary.pdf]

Failure criteria for fiber composite materials considering the effect of material strain rate.

To reduce the error in the numerical simulation results caused by the strain rate effect of fiber materials, this paper takes into account the strain rate effect of fiber materials in the numerical simulation study. Moreover, the strain rate effects in different directions are respectively defined as the dynamic strength factor DIF. DIF is the ratio of the material strength under the actual strain rate to that under the reference strain rate.

$$S = S_0 \times DIF_s = \left[S_{1T}, S_{1C}, S_{2T}, S_{2C}, S_{3T}, S_{3C}, S_{12}, S_{13}, S_{23}\right]^T \tag{S1}$$

$$DIF_s = 1 + R \times \ln\left(\dot{\varepsilon} / \dot{\varepsilon}_0\right) \tag{S2}$$

where:

$\dot{\varepsilon}$ is the strain rate; $\dot{\varepsilon}_0$ represents the reference strain rate, in this study, $\dot{\varepsilon}_0$ is $10^{-4}s^{-1}$; S represents the strength value ; R represents the parameter of strain rate effect.

In this study, the failure of the fiber material is based on the Hashin criterion, and the Yeh layer failure criterion for inter-fiber failure is also incorporated to define the damage evolution of the composite material in the form of strain. The Hashin failure criterion and the Yeh layer failure criterion include the following failure modes:

(a) Fiber tensile ($\varepsilon_{11} > 0$):

$$R_{ft}^{\ 2} = \left(\frac{\varepsilon_{11}}{X_T^{\varepsilon}}\right)^2 \tag{S3}$$

(b) Fiber compression ($\varepsilon_{11} < 0$):

$$R_{fc}^{\ 2} = \left(\frac{\varepsilon_{11}}{X_C^{\varepsilon}}\right)^2 \tag{S4}$$

(c) Matrix tensile ($\varepsilon_{22} + \varepsilon_{33} \geq 0$):

$$R_{mt}^{\ 2} = \left(\frac{\varepsilon_{11} + \varepsilon_{33}}{Y_T^{\varepsilon}}\right)^2 + \left(\frac{1}{S_{23}^{\varepsilon\ 2}}\right)\left(\varepsilon_{23}^{\ 2} - \frac{E_{22}E_{33}}{G_{23}^{\ 2}}\varepsilon_{22}\varepsilon_{33}\right) + \left(\frac{\varepsilon_{12}}{S_{12}^{\varepsilon}}\right)^2 + \left(\frac{\varepsilon_{13}}{S_{13}^{\varepsilon}}\right)^2 \tag{S5}$$

(d) Matrix compression ($\varepsilon_{22} + \varepsilon_{33} < 0$):

$$R_{mc}{}^2=\left(\frac{E_{22}\varepsilon_{22}+E_{33}\varepsilon_{33}}{2G_{12}S_{12}^{\varepsilon}}\right)^2+\left(\frac{\varepsilon_{22}+\varepsilon_{33}}{Y_C^{\varepsilon 2}}\right)\left[\left(\frac{E_{22}Y_C^{\varepsilon}}{2G_{12}S_{12}^{\varepsilon}}\right)^2-1\right]$$

$$+\frac{1}{S_{23}^{\varepsilon\,2}}\left(\varepsilon_{23}{}^2-\frac{E_{22}E_{33}}{G_{23}{}^2}\varepsilon_{22}\varepsilon_{33}\right)+\left(\frac{\varepsilon_{12}}{S_{12}^{\varepsilon}}\right)^2+\left(\frac{\varepsilon_{13}}{S_{13}^{\varepsilon}}\right)^2 \tag{S6}$$

(e) Layered ($\varepsilon_{33}\geq 0$):

$$R_{ld}{}^2=\left(\frac{\varepsilon_{33}}{Z_T^{\varepsilon}}\right)^2+\left(\frac{\varepsilon_{13}}{S_{13}^{\varepsilon}}\right)^2+\left(\frac{\varepsilon_{23}}{S_{23}^{\varepsilon}}\right)^2 \tag{S7}$$

where：

X_T^{ε} , X_C^{ε} are the strains corresponding to the tensile and compressive strengths in the X direction of the fibers respectively;

Y_T^{ε} , Y_C^{ε} are the strains corresponding to the tensile and compressive strengths in the Y direction of the fibers respectively;

S_{12}^{ε} , S_{13}^{ε}, S_{23}^{ε} are the shear strains in different directions of the fibers;

Z_T^{ε} represents the strain corresponding to the tensile strength of the fiber in the direction perpendicular to the fiber surface.

where, $X_T^{\varepsilon}=\frac{X_T}{E_{11}}$, $X_C^{\varepsilon}=\frac{X_C}{E_{11}}$, $Y_T^{\varepsilon}=\frac{Y_T}{E_{22}}$, $Y_C^{\varepsilon}=\frac{Y_C}{E_{22}}$, $S_{12}^{\varepsilon}=\frac{S_{12}}{G_{12}}$, $S_{13}^{\varepsilon}=\frac{S_{13}}{G_{13}}$, $S_{23}^{\varepsilon}=\frac{S_{23}}{G_{23}}$, $Z_T^{\varepsilon}=\frac{Z_T}{E_{33}}$ 。

To assess the degree of damage in composite materials, a damage variable d_i is defined. The domain of the damage variable is [0,1]. When d_i=0.0, no damage occurs; when d_i>0.0, damage begins to appear; when d_i=1.0, the material fails. The damage variable d_i is correlated with the failure factor R_i to determine the failure of the material. The relationship between d_i and R_i is as follows (Equation 8)：

$$d_i=\begin{cases}0 & (R_i<1)\\ 1-\frac{1}{R_i^n} & (R_i\geq 1)\end{cases}\quad (i=ft,fc,mt,mc,ld) \tag{S8}$$

Where, n is a positive quantity with a dimension of 1, which is used to control the rate of stiffness degradation of the material during the damage process. Here, the damage is an irreversible parameter that changes over time, so its relationship with the time t at any moment is:

$$d_i^t = \max\left(d_i^\tau, 0\right), \left(\tau \le t; i = ft,\ fc, mt, mc, ld\right) \tag{S9}$$

The constitutive equation for the material after damage and destruction can be expressed as Equation 10:

$$\begin{Bmatrix} \varepsilon_{11} \\ \varepsilon_{22} \\ \varepsilon_{33} \\ \gamma_{12} \\ \gamma_{23} \\ \gamma_{13} \end{Bmatrix} = \begin{bmatrix} \frac{1}{E_{11}(1-\omega_1)} & -\frac{\nu_{12}}{E_{22}} & -\frac{\nu_{13}}{E_{33}} & 0 & 0 & 0 \\ -\frac{\nu_{12}}{E_{22}} & \frac{1}{E_{22}(1-\omega_2)} & -\frac{\nu_{23}}{E_{22}} & 0 & 0 & 0 \\ -\frac{\nu_{13}}{E_{33}} & -\frac{\nu_{23}}{E_{22}} & \frac{1}{E_{33}(1-\omega_3)} & 0 & 0 & 0 \\ 0 & 0 & 0 & \frac{1}{G_{12}(1-\omega_4)} & 0 & 0 \\ 0 & 0 & 0 & 0 & \frac{1}{G_{23}(1-\omega_5)} & 0 \\ 0 & 0 & 0 & 0 & 0 & \frac{1}{G_{13}(1-\omega_6)} \end{bmatrix} \begin{Bmatrix} \sigma_{11} \\ \sigma_{22} \\ \sigma_{33} \\ \tau_{12} \\ \tau_{23} \\ \tau_{13} \end{Bmatrix} \tag{S10}$$

Where, the damage parameter $\omega_i = \left(i = 1, \cdots, 6\right)$ is defined as:

$\omega_1 = \max\left(0.0, d_f\right)$, $\omega_2 = \max\left(0.0, d_f, d_m\right)$, $\omega_3 = \max\left(0.0, d_f, d_d\right)$

$\omega_4 = \max\left(0.0, d_f, d_m\right)$, $\omega_5 = \max\left(0.0, d_f, d_d\right)$, $\omega_6 = \max\left(0.0, d_f, d_d\right)$

$d_f = \max\left(0.0, d_{ft}, d_{fc}\right)$, $d_m = \max\left(0.0, d_{mt}, d_{mc}\right)$, $d_d = \max\left(0.0, d_{ld}\right)$
